# Clinicopathological and prognostic significance of circulating tumor cells in patients with lung cancer: a meta-analysis

**DOI:** 10.18632/oncotarget.19122

**Published:** 2017-07-10

**Authors:** Tingjuan Xu, Guodong Shen, Min Cheng, Weiping Xu, Gan Shen, Shilian Hu

**Affiliations:** ^1^ Gerontology Institute of Anhui Province, Anhui Provincial Hospital Affiliated Anhui Medical University, Hefei 230001, China; ^2^ Anhui Provincial Key Laboratory of Tumor Immunotherapy and Nutrition Therapy, Hefei 230001, China

**Keywords:** circulating tumor cells, lung cancer, prognosis, clinicopathological parameters, meta-analysis

## Abstract

**Background:**

The prognostic significance of circulating tumor cells in patients with lung cancer is controversial. Therefore, we aimed to comprehensively and quantitatively assess the prognostic role of CTCs in patients with lung cancer.

**Methods:**

The relevant literature was searched using PubMed, the Cochrane database and the China National Knowledge Internet database (up to June 2016). Using Review Manager 5.1.2, a meta-analysis was performed using hazard ratio (HR), odds ratio (OR) and 95% confidence interval (CI) as effect values.

**Results:**

Thirty studies comprising 2,060 patients with lung cancer were analyzed. The pooled HR values showed that circulating tumor cells were significantly correlated with overall survival (HR =2.63, 95% CI [2.04, 3.39]) and progression-free survival (HR =3.74, 95% CI [2.49, 5.61]) in these patients. Further subgroup analyses were conducted and categorized by sampling time, detection method, and histological type; these analyses showed the same trend. The pooled OR values showed that circulating tumor cells were associated with non small cell lung cancer stage(OR = 2.11, 95% CI [1.42, 3.14]), small cell lung cancer stage (OR = 10.91, 95% CI [4.10, 29.06]), distant metastasis (OR =7.06, 95%CI [2.82, 17.66]), lymph node metastasis (OR =2.31, 95% CI [1.19,4.46]), and performance status(OR =0.42, 95%CI [0.22, 0.78]).

**Conclusion:**

The detection of circulating tumor cells in the peripheral blood of patients with lung cancer can be indicative of a poor prognosis.

## INTRODUCTION

Lung cancer is one of the deadliest diseases in the world. Less than 15% of lung cancer patients survive for more than 5 years after being diagnosed [1]. Due to its aggressive behavior and greater invasive ability than other types of cancer, the predominant cause of treatment failure in patients with lung cancer is believed to be distant metastases, even during early-stage disease. Approximately 25% to 50% of patients with early-stage non small cell lung cancer (NSCLC) show tumor recurrence, even after tumor resection [2, 3]. However, current staging methods are unable to detect such occult metastases prior to the emergence of clinical manifestations [4]. Thus, there is an urgent need for more-sensitive prognostic and predictive markers.

Circulating tumor cells (CTCs) can be found in the peripheral blood of patients with cancer. Many studies have demonstrated the potential usefulness of CTCs in predicting patient prognosis for several cancer types [5–7]. Many studies have also shown associations between CTCs and poor survival in lung cancer [8–11]. However, the prognostic significance of CTCs in lung cancer remains controversial, as other studies have failed to show an association between CTCs and poor prognosis [12]. In addition, assessing the potential of using CTCs as a prognostic marker has been complicated by inter-study differences in aspects such as study population, methodology and sampling time.

Thus, our meta-analysis aimed to examine the association of CTCs with survival and clinicopathological parameters, and to evaluate the prognostic role of CTCs in patients with lung cancer.

## RESULTS

### Characteristics of the included studies

**Figure 1:**
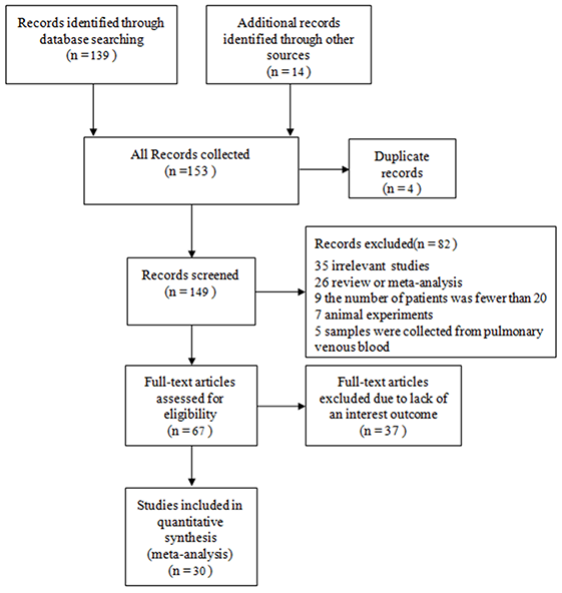
A flow chart of the study design A systematic literature search yielded a total of 153 articles related to the relationship between CTCs and lung cancer. After the screening of titles, abstracts and full texts, 123 articles were excluded for reasons detailed in the main text. A meta-analysis was then performed on 30 studies to assess the clinicopathological and prognostic significance of CTCs in patients with lung cancer.

After initial literature searches, 153 articles were retrieved, and 4 duplicate articles were excluded. After screening the titles and abstracts, 67 studies remained, and their full texts were assessed for eligibility. Of the eligible studies, 37 studies were excluded because they lacked an outcome of interest. Ultimately, 30 studies were selected for analysis; these comprised 24 studies published in English and 6 studies published in Chinese (Figure 1).

The analyzed studies were from the Netherlands, the United Kingdom, America, Spain, Japan, Korea and China, and included a total of 2,060 patients. The median number of patients in each study was 69 (range, 28-208). Of these 30 studies, 2 studies addressed both NSCLC and small cell lung cancer (SCLC); 22 studies addressed NSCLC alone, and 6 addressed SCLC alone. The sampling time was divided into two time points: namely, pre- and post-treatment. Both time points were included in 12 studies [9–11, 13–21], pretreatment alone in 15 studies [8, 12, 22–34] and post-treatment alone in 3 studies [35–37]. Nine studies used the reverse-transcriptase polymerase chain reaction (RT-PCR) method, and 21 studies used other methods. Hazard ratio (HR) for overall survival (OS) and progression-free survival (PFS) could be extracted from 12 studies and 4 studies, respectively. The patient clinical characteristics and the design variables of the studies are summarized in Table 1. The quality of the 30 included studies was evaluated according to the Newcastle-Ottawa scale (NOS) (Table 2). Twenty-five studies were of high quality (NOS score ≥ 5), and 5 studies were of low quality (NOS score < 5).

**Table 1:** Characteristics and design variables of the including studies

Author	country	No. of patients	Age	Histological features	Treatment	Sampling volume	Methods	Markers	Sampling time	Cutoff of CTC
Chen TF *et al*	China	67	62(40-75)	ADC 32 SQC 32 Others 3	chemo. and radio.	8ml	RT-PCR	CK19 mRNA	pre and post	NR
Hiltermann TJ *et al*	Holland	59	64(47-84)	SCLC 59	chemo. and radio.	7.5ml	Cellsearch	EpCAM,CK8,18,19,DAPI	pre and post	2 CTCs
Hofman V *et al*	NR	208	63(37-84)	ADC 115 SQC 54 Others 39	surg.	10ml	ISET	NR	pre	50
Hou JM *et al*	UK	97	68(28-84)	SCLC 97	chemo.	7.5ml	Cellsearch, ISET	EpCAM,CK8,18,19,DAPI	pre and post	50 CTCs
Igawa S *et al*	Japan	30	69(51-85)	SCLC 30	chemo.	7.5ml	IF	GFP	pre and post	2 CTCs
Krebs MG *et al*	UK	101	67(43-84)	ADC 31 SQC 32 Other 38	chemo. and radio.	7.5ml	Cellsearch	EpCAM,CK8,18,19,DAPI	pre and post	2 CTCs
Naito T *et al*	Japan	51	67(34-92)	SCLC 51	chemo. or radio.	7.5ml	Cellsearch	EpCAM,CK8,18,19,DAPI	pre	8 CTCs
Nieva J *et al*	America	28	64(31-82)	ADC 21 SQC 5 Others 2	chemo. or biotherapy	NR	IF	CK 1,4–8,10,13,18,19, DAPI	pre	1CTC
Shi WJ *et al*	China	55	59(41-75)	SCLC 55	chemo.	10ml	RTQ-PCR	CK19 mRNA	pre and post	3.8
Yamashita J *et al*	Japan	103	68(35-83)	ADC 66 SQC 37	surg.	NR	RT-PCR	CEA mRNA	pre and post	NR
Yie SM *et al*	China	143	57(30-84)	ADC 87 SQC 56	surg. or chemo.	2ml	RT-PCR	Survivin mRNA	pre	1.02pg/ml
Yoon SO *et al*	Korea	79	66(42-87)	ADC 45 SQC 27 Others 7	surg.	NR	RT-PCR	TTF-1,CK19 mRNA	pre and post	NR
Juan O *et al*	Spain	37	71(44-85)	ADC 14 SQC 14 Others 9	chemo.	7.5ml	Cellsearch	EpCAM,CK8,18,19,DAPI	pre and post	2 CTCs
Sher YP *et al*	China	54	65(28-81)	ADC 35 SQC 14 Others 5	surg. or chemo.	3-4ml	RT-PCR	keratin 19, Ubiquitin thiolesterase C, HSFIB1	pre	NR
Bayarri-Lara C *et al*	Spain	56	67.4(45-80)	ADC 25 SQC 29 Others 2	surg.	10ml	IF	EGFR,CK	pre and post	NR
Chen X *et al*	China	169	NR	ADC 112 SQC 51 Others 6	NR	7.5ml	Cellsearch	EpCAM,CK8,18,19,DAPI	pre	1CTC
Hirose T *et al*	Japan	33	64(46-74)	ADC 24 SQC 8 Others 1	chemo.	7.5ml	Cellsearch	EpCAM,CK8,18,19,DAPI	pre	1CTC
Ji JL *et al*	China	56	68(38-80)	NSCLC	surg.	2ml	ICC	EpCAM	post	1CTC
Lou JT *et al*	China	33	58(33-76)	ADC 16 SQC 11 Others 6	chemo.	3ml	LT-PCR	CK,FR,DAPI	pre	8.5
Peck K *et al*	China	86	66(26-82)	ADC 47 SQC 17 SCLC 15 Others 7	surg. or chemo. or radio.	3-5ml	RT-PCR	CK19 mRNA	pre	NR
Sheu CC *et al*	China	100	64(37-87)	ADC 72 SQC 28	NR	5ml	RT-PCR	17genes	pre	NR
Wang B *et al*	China	42	68(37-80)	ADC 25 SQC 17	surg.	10ml	ICC	EpCAM	post	1CTC
Wu C *et al*	China	47	NR	ADC 27 SQC 7 SCLC 13	chemo.	7.5ml	IF	CK18,19,DAPI	pre	2CTCs
Xu YH *et al*	China	66	69(34-80)	ADC 35 SQC 31	chemo.	7.5ml	Cellsearch	EpCAM,CK8,18,19,DAPI	pre and post	1CTC
Feng YQ *et al*	China	49	NR	ADC 20 SQC 29	NR	7.5ml	IF	EpCAM,CK,DAPI	pre	1CTC
HuangTH *et al*	China	51	58.6(43-75)	ADC 21 SQC 30	surg. or chemo. or radio.	4ml	ICC	CK	pre	1CTC
Li J *et al*	China	30	67(43-79)	ADC 12 SQC 18	chemo.	7.5ml	IF	CK	pre and post	1CTC
Lin XM *et al*	China	60	56(35-76)	ADC 32 SQC 28	surg.	10ml	ICC	CK	pre	1CTC
Qian Z *et al*	China	35	48(21-69)	SCLC 35	NR	7.5ml	Cellsearch	EpCAM,CK8,18,19,DAPI	pre	1CTC
Zhao SW *et al*	China	35	58(43-80)	ADC 31 SQC 4	surg.	3.2ml	IF	DAPI	post	2CTCs

ADC, adenocarcinoma; SQC, squamous cell carcinoma; SCLC, small-cell lung cancer; chemo., chemotherapy; radio., radiotherapy; surg., surgery; IF, immunofluorescence; ISET, isolation by size of epithelial tumor cells; ICC, immunocytochemistry; pre, pre-treatment; post, post-treatment; NR, not reported

**Table 2:** The assessment of the risk of bias in each Cohort study using the Newcastle–Ottawa scale

Study	Selection(0-4)			Comparablility (0-2)			Outcome(0-3)			Total
	REC	SNEC	AE	DO	SC	AF	AO	FU	AFU	
Chen TF	1	1	1	1	0	0	1	1	1	7
Hiltermann TJ	1	1	1	1	0	0	1	1	1	7
Sher YP	1	1	1	1	0	0	1	1	1	7
Hofman V	1	1	1	1	0	0	1	1	0	6
Hou JM	1	1	1	1	0	0	1	0	1	6
Igawa S	1	1	1	1	0	0	1	0	1	6
Shi WJ	1	1	1	1	0	0	1	0	1	6
Yamashita Y	1	1	1	1	0	0	0	1	1	6
Yie SM	1	1	1	1	0	0	1	1	0	6
Yoon SO	1	1	1	1	0	0	1	1	0	6
Krebs MG	0	1	1	1	0	0	1	0	1	5
Naito T	1	1	1	1	0	0	1	0	0	5
Nieva J	1	1	1	1	0	0	0	0	1	5
Juan O	0	1	1	1	0	0	1	0	1	5
Bayarri-Lara C	1	1	1	1	0	0	0	0	1	5
Chen X	1	1	1	1	0	0	1	0	0	5
Hirose T	0	1	1	1	0	0	1	0	1	5
Ji JL	1	1	1	1	0	0	1	0	0	5
Peck K	1	1	1	1	0	0	1	0	0	5
Sheu CC	1	1	1	1	0	0	1	0	0	5
Wang B	1	1	1	1	0	0	1	0	0	5
Wu C	1	1	1	1	0	0	1	0	0	5
Feng YQ	1	1	1	1	0	0	1	0	0	5
Lin XM	1	1	1	1	0	0	1	0	0	5
Qian Z	1	1	1	1	0	0	1	0	0	5
Lou JT	1	1	1	1	0	0	0	0	0	4
Xu YH	0	1	1	1	0	0	1	0	0	4
Huang TH	1	1	1	1	0	0	0	0	0	4
Li J	0	1	1	1	0	0	1	0	0	4
Zhao SW	1	1	1	1	0	0	0	0	0	4

### The prognostic effect (OS and PFS) of CTC detection

**Figure 2:**
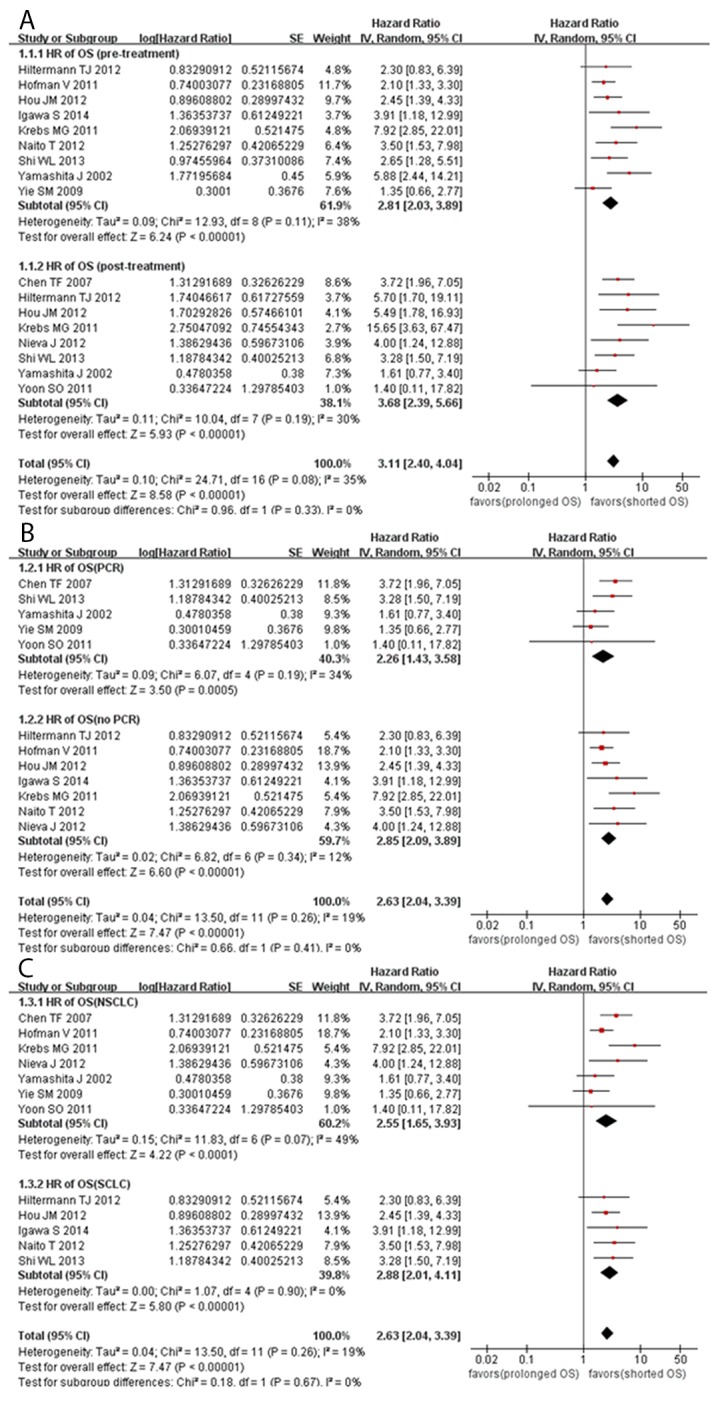
Forest plots evaluating the maximally adjusted association between CTC presence and OS **(A)** A Forest plot assessing the effect of CTC presence on OS in subgroups divided by sampling time. **(B)** A Forest plot assessing the effect of CTC presence on OS in subgroups divided by detection method. **(C)** A Forest plot assessing the effect of CTC presence on OS in subgroups divided by histological type.

The pooled HR values showed a significant correlation between CTCs and OS in patients with lung cancer (HR =2.63, 95% confidence interval (CI) [2.04, 3.39], *P*<0.00001, *I^2^*=19%) (Figure 2). Subsequently, subgroup analyses were conducted after categorization by sampling time, detection method, and histological type to further investigate the prognostic role of CTCs. We found a significant correlation between CTCs and OS in the NSCLC (HR =2.55, 95% CI [1.65, 3.93], *P*<0.0001, *I^2^*=49%) and SCLC subgroups (HR =2.88, 95% CI [2.01, 4.11], *P*<0.00001, *I^2^*=0%). In addition, the results of the analysis showed that CTCs could be a prognostic indicator of OS both pretreatment (HR =2.81, 95% CI [2.03, 3.89], *P*<0.00001, *I^2^*=38%) and post-treatment (HR =3.68, 95% CI [2.39, 5.66], *P*<0.00001, *I^2^*=30%), regardless of whether the RT-PCR method (HR =2.26, 95% CI [1.43, 3.58], *P*=0.0005, *I^2^*=34%) or other methods (HR =2.85, 95% CI [2.09, 3.89], *P*<0.00001, *I^2^*=12%) were used.

**Figure 3:**
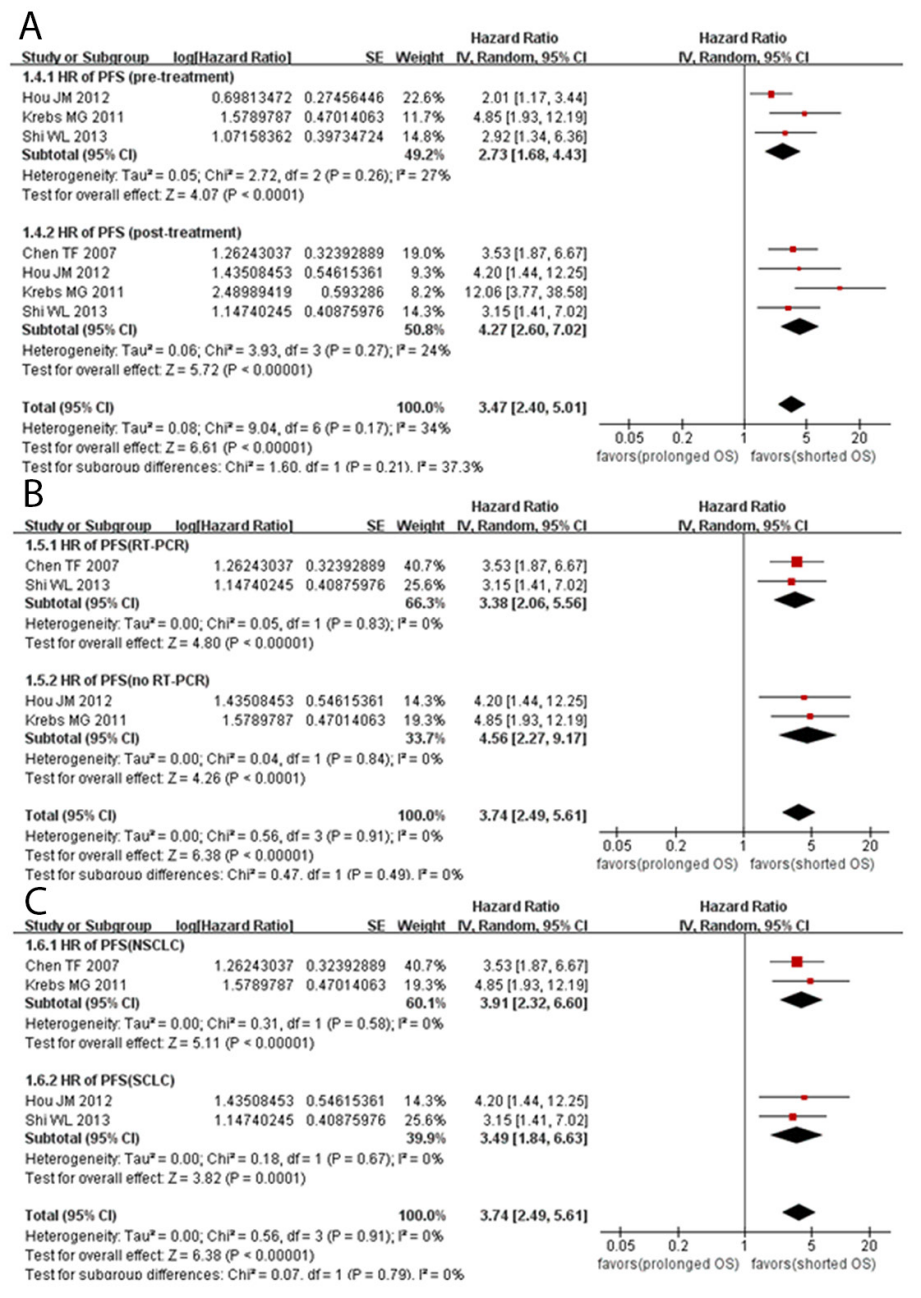
Forest plots evaluating the maximally adjusted association between CTC presence and PFS **(A)** A Forest plot assessing the effect of CTC presence on PFS in subgroups divided by sampling time. **(B)** A Forest plot assessing the effect of CTC presence on PFS in subgroups divided by detection method. **(C)** A Forest plot assessing the effect of CTC presence on PFS in subgroups divided by histological type.

The pooled HR values revealed a significant correlation between CTCs and PFS in patients with lung cancer (HR =3.74, 95% CI [2.49, 5.61], *P*<0.00001, *I^2^*=0%) (Figure 3). The subgroup analyses showed a significant correlation between CTCs and PFS in the NSCLC (HR =3.91, 95% CI [2.32, 6.60], *P*<0.00001, *I^2^*=0%) and SCLC subgroups (HR =3.49, 95% CI [1.84, 6.63], *P*=0.0001, *I^2^*=0%). In addition, we found that CTCs could be a prognostic indicator of PFS both pretreatment (HR =2.73, 95% CI [1.68, 4.43], *P*<0.0001, *I^2^*=27%) and post-treatment (HR =4.27, 95% CI [2.60, 7.02], *P*<0.00001, *I^2^*=24%), regardless of whether the RT-PCR method (HR =3.38, 95% CI [2.06, 5.56], *P*<0.0001, *I^2^*=0%) or other methods (HR =4.56, 95% CI [2.27, 9.17], *P*<0.0001, *I^2^*=0%) were used.

### Correlation between CTCs and clinicopathological parameters

The pooled odds ratio (OR) values showed that there was a significant correlation between CTCs and tumor stage in patients with lung cancer. As shown in Table 3, the incidence of CTC detection in patients with stage III/IV was higher than that in patients with stage I/II NSCLC (OR = 2.11, 95% CI [1.42,3.14], *P*=0.0002, *I^2^*= 20%). Similarly, the incidence of CTC detection in extensive SCLC was higher than that in limited SCLC (OR = 10.91, 95% CI [4.10, 29.06], *P*<0.00001, *I^2^*= 4%). However, the subgroup analyses of studies using the RT-PCR method showed no significant correlation between CTCs and tumor stage in either NSCLC patients or SCLC patients.

**Table 3:** Detailed results of meta-analyses for clinicopathological parameters

Clinicopathological parameters	Sample time	Study no.	Patient no.	OR(95% CI), P	Heterogeneity(I^2^, P)
NSCLC Stage III/IV vs. I/II	overall	15	1123	2.11 [1.42, 3.14], 0.0002	20%, 0.23
	pre	12	990	1.77 [1.17, 2.68], 0.007	16%, 0.29
	post	5	248	3.72 [1.79, 7.72], 0.0004	0%, 0.85
	PCR	7	464	1.25 [0.71, 2.19], 0.44	7%, 0.37
	non-PCR	8	659	2.71 [1.78, 4.13], <0.00001	0%, 0.50
SCLC Extensive vs. Limited	overall	4	202	10.91 [4.10, 29.06], <0.00001	4%, 0.37
	pre	4	202	10.91 [4.10, 29.06], <0.00001	4%, 0.37
	post	1	55	5.75 [1.58, 20.99], 0.008	
	PCR	2	70	6.30 [0.60, 65.68], 0.12	49%, 0.16
	non-PCR	2	132	13.87 [4.30, 44.77], <0.0001	0%, 0.39
Distant metastasis (+) vs. (-)	overall	5	522	7.06 [2.82, 17.66], <0.0001	46%, 0.11
	pre	5	522	7.06 [2.82, 17.66], <0.0001	46%, 0.11
	post	1	55	5.75 [1.58, 20.99], 0.008	
	PCR	2	155	8.58 [2.07, 35.56], 0.003	0%, 0.77
	non-PCR	4	367	7.13 [1.80, 28.21], 0.005	70%, 0.03
	NSCLC	3	370	5.44 [1.40, 21.15], 0.01	45%, 0.16
	SCLC	2	152	11.41 [4.15, 31.39], <0.00001	0%, 0.54
Lymph node metastasis (+) vs. (-)	overall	5	420	2.31 [1.19, 4.46], 0.01	19%, 0.29
	pre	5	420	2.31 [1.19, 4.46], 0.01	19%, 0.29
	post	2	104	1.60 [0.57, 4.46], 0.37	20%, 0.26
	PCR	3	239	2.98 [0.72, 12.29], 0.13	58%, 0.09
	non-PCR	2	181	2.17 [0.97, 4.88], 0.06	0%, 0.60
	NSCLC	5	420	2.31 [1.19, 4.46], 0.01	19%, 0.29
	SCLC	0	0		
Performance status 0-1 vs. 2	overall	4	286	0.42 [0.22, 0.78], 0.006	0%, 0.48
	pre	4	286	0.42 [0.22, 0.78], 0.006	0%, 0.48
	post	1	55	0.85 [0.25, 2.83], 0.79	
	PCR	1	55	0.69 [0.16, 2.96], 0.62	
	non-PCR	3	231	0.37 [0.19, 0.74], 0.005	0%, 0.39
	NSCLC	2	134	0.59 [0.13, 2.80], 0.51	31%, 0.23
	SCLC	2	152	0.38 [0.18, 0.79], 0.01	0%, 0.36
Tumor size (<3cm) vs. (>3cm)	overall	6	445	0.88 [0.55, 1.42], 0.60	0%, 0.54
	pre	4	347	1.06 [0.62, 1.79], 0.83	0%, 0.70
	post	4	202	0.53 [0.27, 1.03], 0.06	0%, 0.75
	PCR	2	161	0.96 [0.39, 2.31], 0.92	10%, 0.29
	non-PCR	4	284	0.84 [0.47, 1.50], 0.56	0%, 0.41
	NSCLC	6	445	0.88 [0.55, 1.42], 0.60	0%, 0.54
	SCLC	0	0		
Gender male vs. female	overall	15	930	1.37 [0.99, 1.89], 0.06	0%, 0.88
	pre	14	1032	1.26 [0.93, 1.70], 0.14	0%, 0.81
	post	5	268	1.21 [0.54, 2.73], 0.64	36%, 0.18
	PCR	5	337	1.29 [0.60, 2.79], 0.51	34%, 0.19
	non-PCR	10	593	1.40 [0.95, 2.06], 0.09	0%, 0.99
	NSCLC	14	875	1.36 [0.97, 1.90], 0.07	0%, 0.84
	SCLC	1	55	1.48 [0.40, 5.50], 0.56	
Age non-aged vs. aged	overall	11	695	0.80 [0.57, 1.13], 0.20	0%, 0.82
	pre	10	653	0.79 [0.56, 1.13], 0.20	0%, 0.75
	post	3	146	1.18 [0.56, 2.48], 0.67	0%, 0.90
	PCR	2	139	0.63 [0.30, 1.33], 0.23	0%, 0.39
	non-PCR	9	556	0.85 [0.58, 1.25], 0.42	0%, 0.79
	NSCLC	11	695	0.80 [0.57, 1.13], 0.20	0%, 0.82
	SCLC	0	0		
Smoking status Never vs. former or current	overall	7	497	0.66 [0.40, 1.07], 0.09	19%, 0.29
	pre	7	497	0.66 [0.40, 1.07], 0.09	19%, 0.29
	post	0			
	PCR	1	54	0.67 [0.20, 2.27], 0.52	
	non-PCR	6	443	0.66 [0.37, 1.17], 0.16	32%, 0.19
	NSCLC	7	497	0.66 [0.40, 1.07], 0.09	19%, 0.29
	SCLC	0	0		

We found that the presence of CTCs was significantly increased in lung cancer patients with distant metastasis (OR =7.06, 95%CI [2.82, 17.66], *P*<0.0001, *I^2^*= 46%). Further subgroup analyses conducted and categorized by sampling time, detection method, and histological type showed the same trend. The presence of CTCs was also significantly increased in lung cancer patients with lymph node metastasis (OR = 2.31, 95%CI [1.19, 4.46], *P*=0.01, *I^2^*= 19%), but the subgroup analyses showed a significant correlation between CTCs and lymph node metastasis only in the pretreatment subgroup. Moreover, all the studies included in this analysis pertained to NSCLC. We also found that CTCs were associated with performance status (OR = 0.42, 95%CI [0.22, 0.78], *P*=0.006, *I^2^*= 0%). Lower performance scores corresponded to lower CTC incidence. However, the subgroup analyses showed no significant correlation in the post-treatment, PCR or NSCLC subgroups.

Furthermore, pooled analyses of tumor size, performance status, smoking status, and patient age revealed no significant correlation between these clinicopathological parameters and CTCs.

### Test of heterogeneity

Except for the ‘non-PCR on distant metastasis’ subgroup (*I^2^*= 70%) and the ‘PCR on lymph node metastasis’ subgroup (*I^2^*= 58%), the heterogeneity among all the included studies was not significant. However, when one study [26] from the ‘non-PCR on distant metastasis’ subgroup was removed, the *I^2^* value was reduced to 0%, while the correlation of CTCs with distant metastasis was unchanged (OR = 14.87, 95% CI [5.00, 44.29], *P*<0.00001). Similarly, when one study [17] from the ‘PCR on lymph node metastasis’ subgroup was removed, the *I^2^* value was reduced to 0%, but the correlation of CTCs with lymph node metastasis was changed (OR = 5.92, 95% CI [1.76, 19.91], *P* =0.004).

### Sensitivity analyses

We performed sensitivity analyses to test the robustness of the pooled results. The pooled HR was not significantly altered when any individual study was removed. Moreover, the pooled OR was not significantly influenced when any individual study was removed, with the exception of lymph node metastasis. The pooled OR of lymph node metastasis was significantly altered by removal of the study [17] that was the source of heterogeneity.

### Publication bias

**Figure 4:**
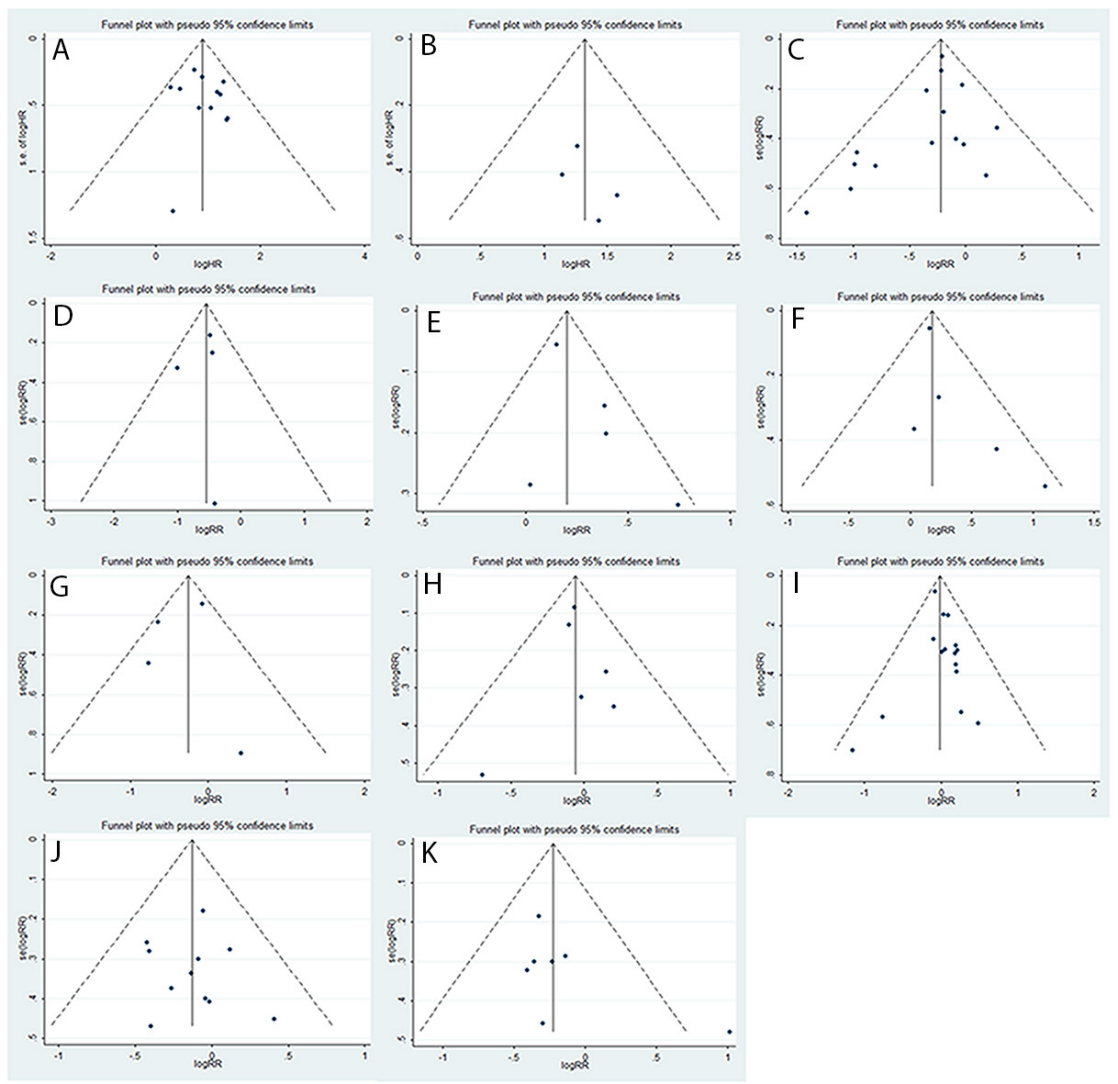
Assessment of publication bias using funnel plot analysis Funnel plot analyses of studies on OS **(A)**, PFS **(B)**, NSCLC stage **(C)**, SCLC stage **(D)**, distant metastasis **(E)**, lymph node metastasis **(F)**, performance status **(G)**, tumor size **(H)**, gender **(I)**, age **(J)** and smoking status **(K)**.

As shown in Figure 4, funnel plots showed no evidence of publication bias. In addition, Egger’s and Begg’s tests were examined to detect publication bias in our article. The results of both Egger’s and Begg’s tests showed no evidence of publication bias (*P*>0.05).

## DISCUSSION

Although chemoradiotherapy and surgery have been widely used, lung cancer metastasis and recurrence frequently occur. The poor overall survival of patients and the complex heterogeneity of the disease are significant challenges for therapeutic intervention. Therefore, biomarkers that can be used to identify lung cancer recurrence or metastasis are needed to facilitate timely diagnosis and effective treatment strategies for lung cancer patients. CTCs, which are released by primary tumors or metastatic tumors, have been recognized as the cause of tumor metastasis or recurrence [38, 39]. However, the clinicopathological and prognostic significance of CTC detection in patients with lung cancer is not clear. In this meta-analysis, we provide strong evidence that CTCs are significantly associated with poor OS and PFS in lung cancer patients, irrespective of sampling time, detection method, and histological type. All the pooled HRs were above 2.0 in our study. These results demonstrate that a CTC-high status indicates poor prognosis in lung cancer patients; these patients may need more-aggressive treatment that is assessed frequently and closely monitored.

According to the pooled ORs in our meta-analysis, CTCs were associated with tumor stage, lymph node metastasis, distant metastasis, and performance status in patients with lung cancer. The results indicated that CTCs can be predictors of disease progression, and may be used to estimate the degree of malignancy and metastatic ability in lung cancer. However, the analysis of lymph node metastasis showed that the correlation occurred only in the pretreatment subgroup. It is generally believed that lymph node metastasis occurs prior to blood-borne metastasis, but the detection of CTCs in patients with early tumors indicates that blood-borne metastasis can occur before lymph node metastasis. In one study [24], the incidence of CTC detection was higher in patients with lymph node metastasis than in those free of lymph node metastasis. However, in other studies [17, 19, 26, 29], the incidence of CTC detection was not correlated with lymph node metastasis. Thus, the correlation between CTCs and lymph node metastasis may require further investigation.

We analyzed studies reporting the detection of CTCs in peripheral blood before and after treatment. The results from these two sampling time were consistent, except for the correlation of CTCs with lymph node metastasis and performance status. Therefore, CTC detection may offer doctors a simpler, less-invasive method that can be used at an earlier stage of disease (relative to other methods) to estimate disease progression and predict the prognosis of patients before treatment.

In recent years, various new CTC assay metho-dologies have been developed, including RT-PCR, immunocytochemistry, and the CellSearch System, for example. Each method has its advantages and disadvantages. We obtained different results for the PCR and non-PCR subgroups in analyses of tumor stage, lymph node metastasis, and performance status. CTCs were not associated with these clinicopathological parameters in the PCR subgroup. It thus seems that non-PCR-based methods are best for CTC detection in this context. Several studies have been performed to compare CTC detection methods, but no conclusive results have been obtained as of yet [40]. Therefore, further studies within the same lung cancer patient populations are needed to provide comparative data on the clinical significance of CTCs detected by different methods.

We found significant heterogeneity in the non-PCR subgroup on distant metastasis and in the PCR subgroup on lymph node metastasis. In these two subgroups, CTCs were detected before treatment using the same detection methods. However, the optimal cut-off values for CTC detection were obviously different for the two subgroups. In addition, the markers of CTC detection were not uniform in the PCR-subgroup studies that investigated the association between CTCs and lymph node metastasis. We propose that these two factors might be the principal causes of heterogeneity.

This study has some notable limitations. First, our meta-analysis was limited to the published scientific literature, and univariate data were also included in the present meta-analysis because multivariate survival analysis data were not available. Second, the CTC detection assays varied in our study, and included different endpoints, cut-off values, and experimental designs. Moreover, we excluded some papers that did not calculate OS and PFS, which may have influenced the results to some degree [18, 25, 41].

In conclusion, this meta-analysis indicates that the detection of CTCs in peripheral blood may be an indicator of patient prognosis, and provides evidence that CTC detection can be used to estimate the degree of malignancy and metastatic ability in patients with lung cancer. In the future, high-quality, well-designed and large-scale multicenter studies are needed to further substantiate these findings.

## METHODS

### Literature search

PubMed, the Cochrane database and the China National Knowledge Internet database were searched for studies pertaining to the clinicopathological and prognostic relationship between CTCs and lung cancer without language, publication or time restrictions (up to June 2016). The main search terms were “lung or pulmonary or pulmonic or pneumonic or pneumal” and “cancer or tumor or tumour or carcinoma or neoplasm(s)” and “CTC(s) or circulating tumor cell(s) or circulating cancer cell(s) or circulating epithelial cell(s) or micrometastasis”. Furthermore, relevant articles were identified from references cited in the retrieved articles and in review articles by manual searching.

### Selection criteria

Eligible studies were included if they met the following criteria: (*i*) CTCs were detected in lung cancer patients; (*ii*) samples were collected from peripheral blood; and (*iii*) at least one of the outcome measures of interest was reported in the study or calculated from published data. When several studies were reported from the same authors or organizations, the meta-analysis included the most recent study (or the highest-quality study if the most recent study did not fit the inclusion criteria).

Studies were excluded if they met any of the following criteria: (*i*) the number of patients with lung cancer was fewer than 20; (*ii*) repeated studies were based on the same database or patients; or (*iii*) they provided insufficient data.

### Data extraction and assessment of study quality

Two independent reviewers evaluated each study and extracted data independently, and any disagreements were resolved *via* discussion. We performed two types of analysis. The first type of analysis determined whether CTC status was associated with OS or PFS. The second type of analysis determined whether CTC status was correlated with clinicopathological parameters, which included tumor size, lymph node metastasis, distant metastasis, NSCLC stage(III/IV vs. I/II), SCLC stage (extensive disease vs. limited disease), gender, age, smoking and performance status. Data for multivariate survival analyses reported in the included articles were included in this meta-analysis. If these data were not available, then univariate analytical data were included. The quality of studies was evaluated according to the NOS [42], and studies with an NOS score≥ 5 were considered to be of high quality.

### Statistical analysis

Statistical analysis was performed using Review Manager 5.1.2software. The estimated HR was used to evaluate the prognostic effect (OS and PFS), and the estimated OR was used to summarize the association between CTC detection and the clinicopathological characteristics of lung cancer. If the HR and its variance were not reported directly in the original study, then these values were calculated from the available reported data using software designed by Tierney *et al.* [43]. All statistical values were combined with a 95% CI, and the *P*-value threshold was set at 0.05. The random-effects mode was used to perform the analysis, as this model produced more conservative results than did the fixed-effects model, and it was a better fit for the multicenter clinical studies owing to the existence of heterogeneity [44]. Heterogeneity was calculated using a Q test, and the *I*^2^ value represented the degree of heterogeneity. Publication bias was tested using a funnel plot, and by Egger’s and Begg’s tests, in Stata 12.0 software. The overall analysis was completed by evaluating all the relevant studies according to different clinicopathological parameters and prognostic outcomes. Further subgroup analyses were conducted and categorized by sampling time (pretreatment and post-treatment), detection method (PCR and non-PCR), and histological type (NSCLC and SCLC). Sensitivity analyses were performed by excluding one study at a time to evaluate the influence of single studies on summary effect values.

## Abbreviations

CI: confidence intervals
CTCs: circulating tumor cells
HR: hazard ratio
NOS: Newcastle-Ottawa scale
NSCLC: non small cell lung cancer
OR: odds ratio
OS: overall survival
PFS: progression-free survival
RT-PCR: reverse-transcriptase polymerase chain reaction
SCLC: small cell lung cancer
